# The second shot to walled‐off necrosis: The sooner the better versus sooner or later

**DOI:** 10.1002/deo2.182

**Published:** 2022-11-02

**Authors:** Tatsuya Sato, Ichiro Yasuda, Yousuke Nakai, The WONDERFUL study group in Japan

**Affiliations:** ^1^ Department of Gastroenterology, Graduate School of Medicine The University of Tokyo Tokyo Japan; ^2^ Third Department of Internal Medicine University of Toyama Toyama Japan; ^3^ Department of Endoscopy and Endoscopic Surgery The University of Tokyo Hospital Tokyo Japan

**Keywords:** acute necrotizing, endosonography, pancreatitis, self‐expandable metallic stents

To the Editor,

We read with great interest the article by Pawa et al.,^1^ reporting a retrospective study comparing immediate and delayed direct endoscopic necrosectomy (DEN) after lumen‐apposing metal stent placement for walled‐off necrosis. The authors reported preferable outcomes of the delayed DEN in terms of length of stay and the number of DEN sessions as well as comparable rates of clinical success and adverse events.

There has been a debate about the optimal timing of DEN during the treatment course of walled‐off necrosis.^2^ The rationale of delaying interventions has been supported by a randomized trial by the Dutch pancreatitis group, which demonstrated the potential of delayed catheter drainage in avoiding a part of adjunctive interventions.^3^ However, other groups reported conflicting results that a reduction in an interval until the second intervention might be translated into that in the overall treatment duration.^4^ In the current study, the delayed DEN group consisted of patients presenting with systemic inflammatory response syndrome for whom DEN was postponed for at least 7 days. Since patients with systemic inflammatory response syndrome were generally associated with worse clinical outcomes, we believe that the study results in favor of delayed DEN were promising. Nonetheless, given the time to DEN of more than 7 days in the delayed DEN group, length of stay (reported to be 3 days in this group) might not be a good surrogate of treatment effectiveness. We therefore ask the authors to provide other metrics such as time to clinical success and the rate of avoiding DEN in the delayed group, which would increase the clinical relevance of their findings.

In summary, along with upcoming results of randomized trials by our group (NCT05451901) and others, the current study would help us to address clinical unmet needs in endoscopic treatment of symptomatic walled‐off necrosis and optimize treatment strategies.

## CONFLICTS OF INTEREST

The authors declare that they have no conflicts of interest. Ichiro Yasuda serves as an Associate Editor of *DEN Open*, and Yousuke Nakai serves as an Associate Editor of *Digestive Endoscopy*.

## FUNDING INFORMATION

Japanese Foundation for Research and Promotion of Endoscopy (#1015).

## References

[1] Pawa R , Dorrell R , Clark C , Russell G , Gilliam J , Pawa S . Delayed endoscopic necrosectomy improves hospital length of stay and reduces endoscopic interventions in patients with symptomatic walled‐off necrosis. DEN Open 2023; 3: e162. 36090191 10.1002/deo2.162 PMC9453323

[2] Yasuda I , Takahashi K . Endoscopic management of walled‐off pancreatic necrosis. Dig Endosc 2021; 33: 335–41. 32306430 10.1111/den.13699

[3] Boxhoorn L , van Dijk SM , van Grinsven J *et al.* Immediate versus postponed intervention for infected necrotizing pancreatitis. N Engl J Med 2021; 385: 1372–81. 34614330 10.1056/NEJMoa2100826

[4] Yan L , Dargan A , Nieto J *et al.* Direct endoscopic necrosectomy at the time of transmural stent placement results in earlier resolution of complex walled‐off pancreatic necrosis: Results from a large multicenter United States trial. Endosc Ultrasound 2019; 8: 172–9. 29882517 10.4103/eus.eus_108_17 PMC6590004

